# Proper network randomization is key to assessing social balance

**DOI:** 10.1126/sciadv.adj0104

**Published:** 2024-05-03

**Authors:** Bingjie Hao, István A. Kovács

**Affiliations:** ^1^ Department of Physics and Astronomy, Northwestern University, Evanston, IL 60208, USA.; ^2^ Northwestern Institute on Complex Systems, Northwestern University, Evanston, IL 60208, USA.; ^3^ Department of Engineering Sciences and Applied Mathematics, Northwestern University, Evanston, IL 60208, USA.

## Abstract

Social ties, either positive or negative, lead to signed network patterns, the subject of balance theory. For example, strong balance introduces cycles with even numbers of negative edges. The statistical significance of such patterns is routinely assessed by comparisons to null models. Yet, results in signed networks remain controversial. Here, we show that even if a network exhibits strong balance by construction, current null models can fail to identify it. Our results indicate that matching the signed degree preferences of the nodes is a critical step and so is the preservation of network topology in the null model. As a solution, we propose the STP null model, which integrates both constraints within a maximum entropy framework. STP randomization leads to qualitatively different results, with most social networks consistently demonstrating strong balance in three- and four-node patterns. On the basis our results, we present a potential wiring mechanism behind the observed signed patterns and outline further applications of STP randomization.

## Teaser

The underlying principles of social networks are found through a network randomization method.

## INTRODUCTION

Individuals within society can be viewed as nodes in a social network, with edges representing various relationships between them. These relationships are highly diverse in their nature and can often be expressed in either positive (friend/trust) or negative (foe/distrust) terms (*1*), leading to signed social networks, with varying degrees of polarization (*2*, *3*). Quantifying the abundance of network patterns is the first step toward understanding why certain connections are formed and not others, captured by the underlying wiring mechanisms, as well as toward understanding and potentially reducing polarization in social media (*2*, *4*–*8*). As a key concept, network graphlets (and motifs) (*9*, *10*) are patterns of connections that occur significantly more frequently than in a null model, which is a suitably randomized version of the empirical data (*9*). Graphlets, also known as induced subgraphs (*11*), specify the existence and sign of every edge within a subset of nodes. In contrast, motifs (or noninduced subgraphs) (*9*), specify only the required edges, allowing for the presence or absence of other edges. For instance, in an undirected signed network, a graphlet consisting of three nodes connected by two edges indicates the absence of the third edge, while a motif detects instances both with or without the third edge. Note that any fully connected graphlet can be equivalently referred to as a motif.

Seminal studies have shown that network graphlets and motifs play an important role in understanding the organization, functionality, and hidden mechanisms behind many complex systems, from social networks to brain connectivity and protein-protein interaction networks (*9*–*17*). Fully connected “triangle” graphlets of three nodes are particularly informative on tie formation mechanisms between acquaintances of the same node. As a starting point, strong balance (SB) (*18*) captures the intuitive notions of “the friend of my friend is my friend,” “the enemy of my friend is my enemy,” and “the enemy of my enemy is my friend.” All these examples correspond to balanced cycles (a path that starts and ends at the same node) of length three, where the product of edge signs along the cycle is positive. The notion of SB has been extended to cycles of any length, stating that a network is maximally balanced if all cycles are balanced (*18*). In practice, there are often deviations from maximal balance (*19*), requiring the statistical analysis of the enrichment of the studied patterns versus a null model. A null model of statistical power is a randomized network that is as close to the real network as possible without capturing the actual wiring mechanisms. Although it is generally believed that social networks tend to be in somewhat balanced states (*20*, *21*), the conclusions about balance strongly depend on the studied datasets and the chosen null model (*22*–*25*).

As a basic example, the “rewire” null model (*23*) swaps edges between nodes while preserving the node degree (*k*, number of neighbors), leading to networks with disrupted topology. Hence, the conclusions based on the rewire null model mix the pattern formation mechanisms arising from edge signs with those of purely topological origin. For example, an overrepresentation of certain patterns might stem solely from the observation that those patterns have a low probability of forming at a purely topological level, regardless of the edge signs.

**Fig. 1.**
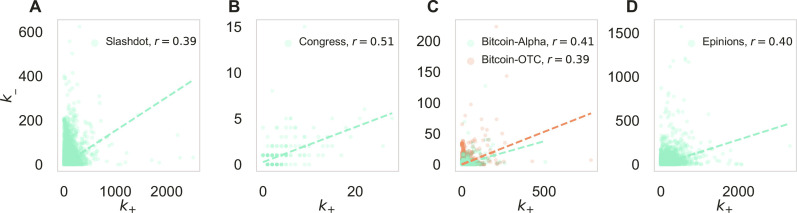
Signed degree correlations. The positive (*k*_+_) and negative (*k*_−_) node degree correlation in (**A**) Slashdot, (**B**) Congress, (**C**) Bitcoin-Alpha, and Bitcoin-OTC (**D**) Epinions. The *r* values denote the Pearson correlation coefficient between *k*_+_ and *k*_−_ of each dataset, indicating a moderate correlation between *k*_+_ and *k*_−_. The dashed line represents the linear fit.

Here, we aim to disentangle purely topological effects from mechanisms of balance related to edge signs by fixing the topology while randomizing the edge signs. As a realization, a more commonly used null model that preserves the network topology is the “sign shuffle” null model (*22*). In this null model, the total number of positive and negative edges is exactly preserved, while the sign is randomly assigned to each edge. Note that the sign shuffle null model has the limitation that all nodes are assumed to have the same expected ratio of positive edges. As illustrated in Fig. 1, this assumption is far from reality. In real-life networks, some nodes are more “friendly” (“hostile”) than others, i.e., holding mostly positive (negative) edges. Consequently, null models that neglect the signed node degree could yield biased conclusions regarding balance.

To incorporate both insights, a null model that preserves both the network topology and signed node degree is needed. State-of-the-art null models only preserve one of these constraints (*22*, *23*, *26*). As a solution, we propose an alternative null model, a signed degree and topology preserving (STP) null model based on the maximum entropy framework (*27*–*31*). The STP null model preserves the network topology exactly, while also matching the signed node degrees on average (see Materials and Methods).

We examine the signed network patterns on a collection of signed social networks covering datasets of various scales, including (i) Slashdot, a friend/foe network in the technological news site Slashdot (*1*); (ii) Congress, a political network where signed edges represent (un/)favorable interactions between US congresspeople on the House floor in 2005 (*32*); (iii) Bitcoin-Alpha, a trust/distrust network of Bitcoin traders on the platform Bitcoin Alpha (*33*); (iv) Bitcoin-OTC, a trust/distrust network of Bitcoin traders on the platform Bitcoin OTC (*34*); and (v) Epinions, a trust/distrust network among users of the product review site Epinions (*1*). For an overview of these datasets, see Table 1. As a key motivation for our work, we observe that positive (*k*_+_) and negative (*k*_−_) node degrees are at best moderately correlated in the studied networks (Fig. 1), indicating that null models that do not consider the signed degree as a confounding factor may lead to biased results. As a key result, we show that, apart from the STP null model, none of the studied null models detect SB even in a simple reference network (SB reference), which is explicitly designed to exhibit SB. In the example of large-scale signed social networks, we show that the STP null model changes the results qualitatively, leading to a consistent interpretation of signed patterns. We conclude by discussing potential underlying pattern formation mechanisms behind our observations, as well as further applications and extensions of STP randomization.

**Table 1.** Overview of studied networks.

Dataset	Slashdot	Congress	Bitcoin-Alpha	Bitcoin-OTC	Epinions	SB ref	EC ref
Nodes	82,052	219	3766	5857	119,070	120,000	120,000
Edges	498,527	520	13,872	21,131	701,569	547,868	790,591
Density	0.00015	0.02178	0.00196	0.00123	0.00010	0.00008	0.00011
Positive ratio	0.76411	0.79615	0.91703	0.86271	0.83215	0.82187	0.72319

## RESULTS

### Signed null models

**Fig. 2.**
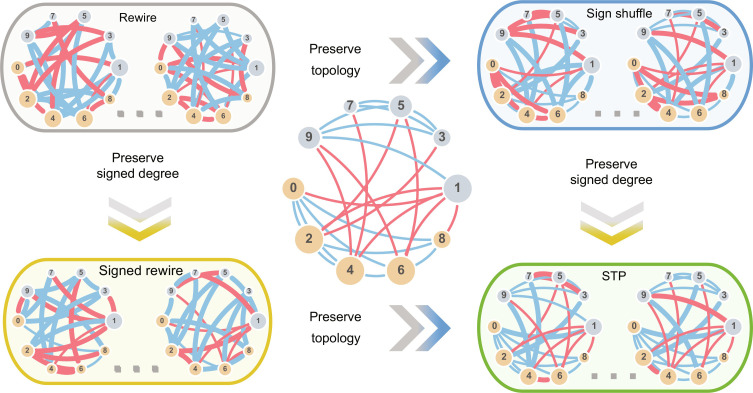
Overview of signed null models. A small toy network that contains two groups of nodes (even in yellow and odd in gray) is shown in the middle. The network is designed to be strongly balanced with positive edges only between members of the same group and negative edges only between members of different groups. We consider four null models: (i) rewire, disrupts the topology and signed node degrees; (ii) sign shuffle, preserves the topology, disrupts signed node degrees; (iii) signed rewire, preserves signed node degrees, disrupts the topology; and (iv) STP, preserves both the topology and signed node degrees. Positive edges are shown in blue, while negatives are in red. Thicker lines indicate edges that are different from the original network.

To investigate how the topology and signed degree affect the graphlet statistics, we consider four null models for signed networks, see Fig. 2. In addition to the commonly used rewire and sign shuffle null models and our STP null model, we also consider the “signed rewire” null model (*35*). The signed rewire null model preserves the signed node degree by rewiring the positive and negative subgraphs separately. As a result, it preserves the signed node degrees while disrupting the topology. In Fig. 2, we illustrate the studied null models on a toy network satisfying SB. This toy network contains two groups of nodes (indicated by different node colors), with positive edges among group members and negative edges between the groups (*36*). Note that some nodes are more friendly (like node 0) or more hostile (like node 1), i.e., have a higher fraction of positive (or negative) edges than others.

### Signed triangle patterns

**Fig. 3.**
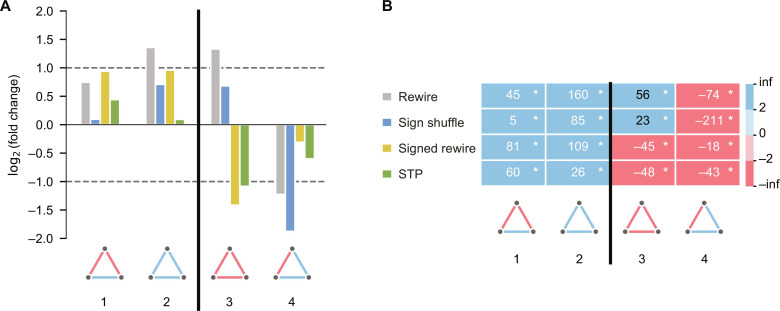
Signed triangles in the Slashdot network compared to different null models. (**A**) The log_2_(fold change) is shown with gray dashed lines indicating a twofold increase or decrease. (**B**) *z* scores are shown in white if matching SB expectations and in black otherwise. The background of the *z* scores is blue for positive values and red for negative values. We list the balanced graphlets first, separated from the unbalanced graphlets by a black line. * marks significant results with ∣*z*∣ > 2 and *P* < 0.01. The statistical analysis is performed using a sample size of *n* = 1000.

**Fig. 4.**
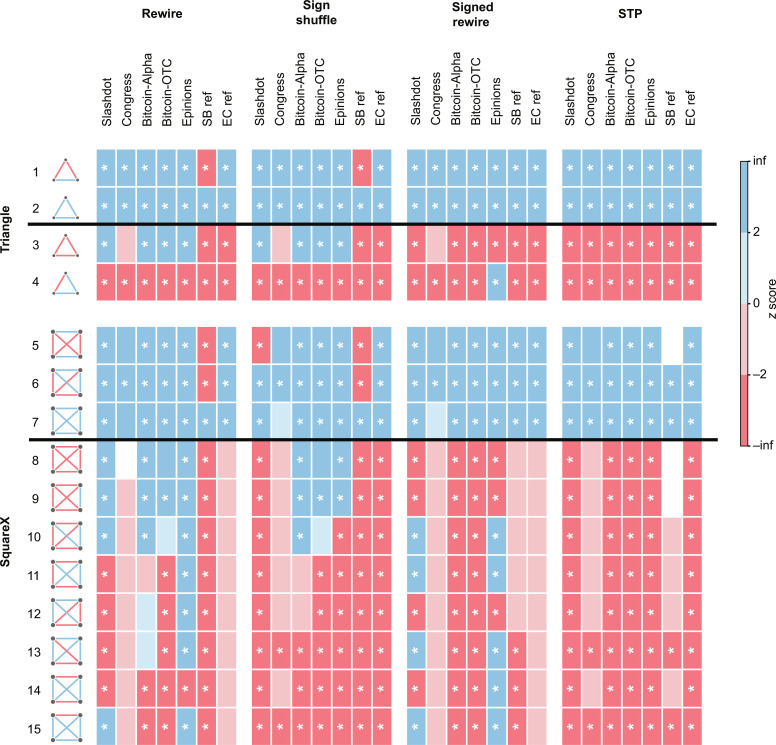
Overview of the results for fully connected graphlets/motifs. The *z* scores are indicated by blue (overrepresented) and red (underrepresented) blocks. We list the balanced graphlets first, separated from the unbalanced graphlets by a black line. We leave the block white if σ_rand_ = 0 as it leads to an undetermined *z* score. Significant results with both ∣*z*∣ > 2 and *P* < 0.01 are indicated by *. The statistical analysis is performed using a sample size of *n* = 1000.

To test social balance in real networks, we first consider the signed fully connected three-node graphlets, triangles, as illustrated in Fig. 3A for the Slashdot network. Each triangle graphlet is counted (*n*_obs_) and compared to the frequency distribution of such triangle (*n*_rand_) in the four null models. We first perform a normality test (*37*) for the null model frequencies of each graphlet *n*_rand_ and achieve *P* > 0.05 for most cases. This implies the lack of substantial evidence to reject the null hypothesis that *n*_rand_ conforms to a normal distribution (fig. S1). This is expected, as *n*_rand_ is the sum of several almost independent random variables. Thus, we use the routinely applied *z* score as a measure to assess the enrichment or depletion of graphlets, computed as

$$z=\frac{n_{\text{obs}}-<n_{\text{rand}}>}{\sigma_{\text{rand}}}$$ (1)

where <*n*_rand_> and σ_rand_ denote the mean and SD of *n*_rand_ in 1000 random samples, respectively. To assess the significance of the results, we calculate the empirical *P* value in Figs. 3 and 4 (see Materials and Methods for details). We only interpret significant results with *P* < 0.01 and ∣*z*∣ > 2. In addition, we also calculate the fold change = *n*_obs_/<*n*_rand_> to indicate the relative abundance of the studied patterns.

In the Slashdot network, both the rewire and sign shuffle null models would conclude that only + + − triangles are underrepresented (Fig. 3A). This conclusion aligns with the notion of weak balance (WB) (*1*, *38*, *39*). WB relaxes the notion of balance so that only triangles with exactly one negative edge should be underrepresented. On the contrary, the signed rewire and STP null models identify that both − − − and + − − triangles are underrepresented, in line with SB. To gain more insight, we benchmark the performance of different null models by constructing a simple SB reference network (*40*) that is designed to exhibit SB (see details in Materials and Methods). As a clear limitation, the rewire and sign shuffle null models fail to detect SB even in the SB reference network (Fig. 4), as they mistakenly identify the + − − triangles as being underrepresented. This observation indicates the essential role of matching the heterogeneous signed degrees in the null models, narrowing down suitable null models to the signed rewire and STP models.

With the STP null model, we observe significant SB in all studied datasets at the triangle level (Fig. 4). The results of the signed rewire null model are again consistent with SB, apart from the Epinions dataset, where the + + − pattern appears to be overrepresented. To underline the importance of reference datasets, the rewire and sign shuffle null models appear to be consistent with WB in all real networks, a disturbingly misleading result, as these null models fail to detect SB even in the SB reference dataset. To sum up, null models that disrupt the signed degree preferences lead to erroneous conclusions at the triangle level. This incoherence is further amplified when analyzing four-node graphlets, as discussed next.

### Signed four-node patterns

Four-node graphlets are useful in uncovering higher-order structures and assessing network reliability across various fields (*41*–*43*). However, as we will demonstrate, when analyzing social networks, comparing the observed frequencies of signed four-node graphlets to existing null models often yields inconsistent conclusions about structural balance across datasets. This again highlights the need for a proper null model that can help uncover the effect of balance in social networks. Just like for three-node graphlets, we start by considering fully connected four-node graphlets, squareX (patterns 5 to 15 in Fig. 4). We will then discuss four-node graphlets that are missing either one (squareZ) or both (square) diagonal edges. We define these graphlets to be balanced if all the cycles within the graphlet are balanced. Just like at the triangle level, the rewire and sign shuffle null models fail to detect SB at the squareX level even in the SB reference network (balanced patterns 5 to 6 appear to be underrepresented), rendering them unsuitable for our purposes (Fig. 4). In terms of the real-life datasets, the picture appears to be rather confusing, as each of the unbalanced squareX graphlets can be either significantly over- or underrepresented depending on the choice of network data and the null model (Fig. 4). In stark contrast, with the STP null model, the results are consistent with SB. The signed rewire null model again leads to inconsistent results across the datasets, as in addition to the Epinions dataset, Slashdot also appears to violate SB for multiple graphlets.

**Fig. 5.**
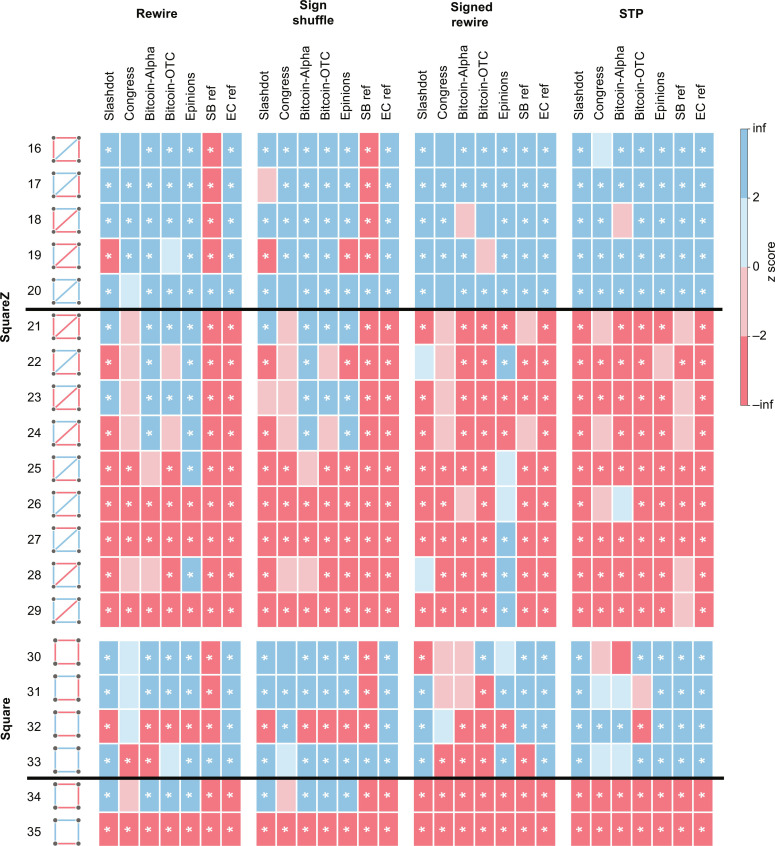
Overview of the results for four-node graphlets with some edges missing (squareZ and square). The *z* scores are indicated by blue (overrepresented) and red (underrepresented) blocks. We list the balanced graphlets first, separated from the unbalanced graphlets by a black line. We leave the block white if σ_rand_ = 0 as it leads to an undetermined *z* score. Significant results with both ∣*z*∣ > 2 and *P* < 0.01 are indicated by *. The statistical analysis is performed using a sample size of *n* = 1000.

Since squareX graphlets are considered to be combinations of triangles, it is natural to expect that squareX graphlets are balanced if triangles are balanced. It then looks surprising that the signed rewire model fails to detect SB for squareX patterns in Slashdot, in contrast to the observed SB at the triangle level. This is an example that even for graphlets composed of triangles, the results do not simply follow from those for triangles. The reason is that significant SB at the triangle level still often comes with a considerable number of unbalanced triangles, contributing in a nonlinear way to the statistics of squareX and squareZ graphlets. Yet, four-node graphlets with fewer constraining edges may exhibit even less balance, calling for the analysis of squareZ (graphlets 16 to 29) and square (graphlets 30 to 35) patterns (Fig. 5). With the STP null model, squareZ graphlets again show consistency with SB (Fig. 5). Just like before, the rewire and sign shuffle null models fail for the SB reference network. At the same time, the signed rewire model deviates from SB only for the Epinions dataset.

Square graphlets can provide additional information as they are not constrained by triangle statistics. In this case, the signed rewire null model also fails to detect SB in the SB reference network, in addition to the rewire and sign shuffle models. Specifically, the + + + + graphlet appears to be significantly underrepresented according to the signed rewire null model (Fig. 5), leaving the STP null model as the only viable null model.

Although there is no anticipation of SB at the square level from the triangle results, we observe significant SB in most of the studied datasets when compared to the STP null model (Fig. 5). The two potential exceptions are the financial datasets, namely, the significant underrepresentation of + − + − in Bitcoin-OTC and the depletion of − − − − in Bitcoin-Alpha, although this deviation is not significant. Note that most of the motif results align with the corresponding graphlet results for the STP null model (fig. S3). The only two exceptions are: − − − − in Epinions, which becomes significantly underrepresented, and + − + − in Bitcoin-OTC, which becomes significantly overrepresented. The observed cases of SB at the square level (with STP null model) call for an interpretation, independently from patterns at the triangle level. When network topology and node preferences are considered, square-level balance reveals additional balance not captured by triangle-level balance alone, as follows. (i) If two nodes have a shared enemy (friend), they may have more shared enemies (friends), corresponding to + + + + and − − − −; (ii) if two nodes have one shared enemy (friend), they may have more shared friends (enemies), corresponding to + + − −; and (iii) if two nodes have opposite attitudes toward a common neighbor, they may hold opposite attitudes toward more neighbors, corresponding to + − + −.

In addition, we have checked the performance of the null models on randomly rewired SB reference networks, where SB is intentionally reduced (fig. S2A). As expected, none of the null models detect SB or WB in this case for triangles and squares. However, the signed rewire null model generally detects larger *z* scores in these randomized networks than the STP null model. As an even more extreme test, we also considered reversing the signs of the SB reference network, leading to the network “SB rev” (fig. S2B). No method finds SB or WB in this case, and only the STP null model is consistent with the intended structure. Once again, we conclude that matching the topology and the signed degree sequence in the null model is important. Now that we have established the STP null model as the only suitable null model, we turn to discuss some of the potential wiring mechanisms behind our observations with STP.

### Potential mechanisms behind signed patterns

**Fig. 6.**
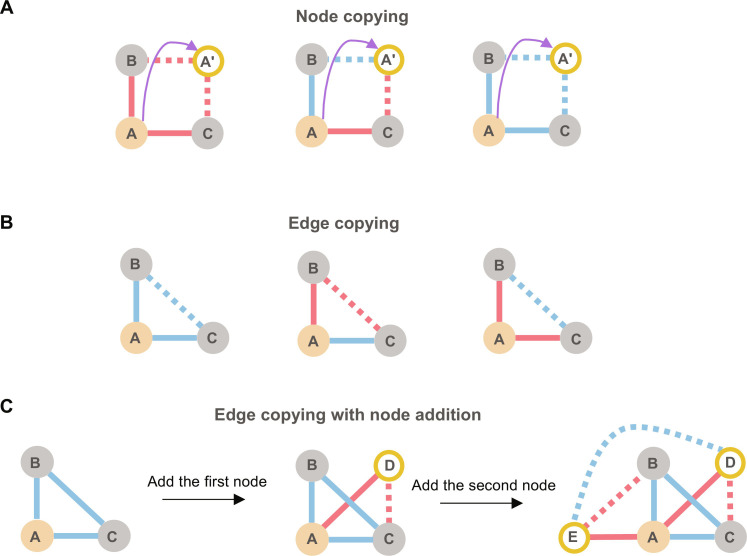
Illustration of signed copying mechanisms. (**A**) Signed node copying. Node *A*′ copies the edges and signs from node *A*, forming balanced squares except the + − + − square. (**B**) Edge copying. Existing connected nodes copy each other’s edges with preserved signs (if positively connected) or reversed signs (if negatively connected). (**C**) Edge copying with node addition. New nodes are added to the network and copy all or some of the edges from a connected node following the edge-copying rules. This process eventually forms triangles, squares (including the + − + − square *B* − *C* − *D* − *E* − *B*), squareZs, and squareXs. Blue lines indicate positive edges and red lines indicate negative edges. Copied edges are shown in dashed lines.

As a starting point, ideas of node-copying mechanisms have been proposed to potentially explain network patterns, including the formation of square graphlets (*44*–*47*). Here, we first generalize the node-copying mechanism to signed networks, where a new node can replicate (some or all) of the connections of another node, also copying the corresponding edge signs. As illustrated in Fig. 6A, when a node (*A*′) duplicates the edges along with their associated signs from another node (*A*), it naturally leads to some of the balanced squares: − − − −, + + − −, and + + + +. Note that the + − + − pattern cannot be created this way. This is in line with the Bitcoin-OTC dataset, where SB is detected apart from the underrepresented + − + − pattern. Yet, all other results in Fig. 5 show an overrepresentation of + − + − graphlets, calling for mechanisms that can potentially explain it. Moreover, signed triangle graphlets are not necessarily explained by a node-copying mechanism, depending also on the initial conditions.

Thus, as an alternative solution, we propose a simple edge-copying mechanism (Fig. 6B). Here, in each step, a node can copy all or some of the edges from its neighbor. Nodes connected by positive edges are assumed to copy each other’s attitudes toward other nodes, just like in the node-copying mechanism. A key difference is that negatively connected nodes are proposed to replicate the edges of their foes with signs reversed. The edge-copying mechanism initially leads to balanced triangles as shown in Fig. 6B and eventually leads to larger balanced graphlets (fig. S4). We implement the proposed edge-copying mechanism together with node addition to generate a simple edge-copying reference network, “EC ref” (see Materials and Methods). The EC reference network starts with a + + + triangle, with nodes sequentially added to the network (see examples in Fig. 6C and fig. S5), and eventually leads to all possible balanced patterns, including the + − + − square graphlet that is missing from the node-copying mechanism. This simple EC reference network only includes balanced patterns (see the proof in the Supplementary Materials) and thus all unbalanced patterns are underrepresented when compared to all null models.

## DISCUSSION

Graphlet (or motif) statistics provide key insights into the mechanisms of network wiring and function. However, it is important to interpret the results in the context of an adequate null model. Up until now, signed network null models had a crucial shortcoming as they either ignored the signed degree preferences of the nodes or the network topology. First, we have shown that matching the signed degrees is critically important in heterogeneous signed networks. Second, we have shown that keeping the network topology intact is useful to disentangle purely topological effects from those related to the balance of signed patterns. As a solution, we proposed the STP null model that preserves both the signed degrees and the network topology using the maximum entropy framework. We found that the STP null model provides more consistent results across signed social networks than previous null models, favoring SB at both the level of triangles and four-node graphlets, with the potential exception of the + − + − graphlet for Bitcoin-OTC. The results suggest that, only when the network topology and node sign preferences are considered, real social networks exhibit a preference for balanced patterns while avoiding unbalanced patterns. This prevalent signature of SB is obscured when topology or signed node degrees are not properly incorporated into the null models. More broadly, the results also highlight the importance of the space of allowed connections. Assuming all connections are possible drastically changes interpretations from a fixed topology. In reality, the topology is neither completely arbitrary nor fixed. Gathering information on the structure of allowed connections would be useful for further understanding the wiring mechanisms of real-world social networks. The STP model could readily incorporate such data on the allowed connections as hard constraints.

In addition, we have introduced an edge-copying mechanism that has the potential to form balanced triangles and four-node graphlets, while providing flexibility in matching the (un)balanced square patterns. Note that the edge-copying mechanism provides a simple, yet plausible, example of forming balanced patterns at the levels of triangles, squareZ and squareX graphlets, questioning the current paradigm that ignores four-node graphlet mechanisms (*36*). Even so, without following the detailed dynamical processes of these networks, we cannot conclude that the edge-copying mechanism is actually at play in these networks. As a complication, when introducing a new node to the network, it can potentially engage in the formation of multiple graphlets simultaneously, both with and without participating in higher-order interactions (*48*, *49*). Furthermore, both node-copying and edge-copying mechanisms may happen simultaneously in real social networks. Note that signed node copying is more plausible when a node can access information on the signed edges of other unrelated nodes, like in the Slashdot and Bitcoin-OTC datasets. Under other circumstances, for online social networks (*50*), individuals may have access to strangers’ friend lists but may have at most limited access to strangers’ blacklists (or foes). Therefore, we expect that the observed patterns might depend on factors like the feasibility and accessibility of copying strangers’ edges. Yet, the consistent SB observed in most datasets indicates a potentially widespread common mechanism, such as edge copying. Any exception detected in the square results may offer clues to understand key aspects of user behavior across platforms. For example, the underrepresentation of + − + − in the Bitcoin-OTC dataset might support the hypothesis that rather than reversing signs from distrustful users, users may decide not to copy those edges instead, leading to fewer + − + − squares.

Here, our primary narrative was to identify null models that are as close to the real data as possible without capturing the wiring mechanisms. As a key step, we proposed to disassociate the purely topological effects from those related to social balance. A substantial difference from a signed null model is then informative on the mechanisms related to balance. Note, however, that this is not the only narrative to consider. The presented null models are also valuable ingredients of an alternative framework where the aim is to match the mechanisms behind real data as closely as possible by the null models. In this sense, the rewire null model corresponds to the scenarios, where individuals can choose both whom they interact with and how. On the other hand, the sign shuffle null model reflects situations where individuals cannot choose whom they interact with but they can decide on how, as long as they have no heterogeneous sign preferences. In situations matching the signed rewire null model, individuals can choose whom to interact with, but with various tendencies of forming positive or negative edges. Last, the STP null model would capture scenarios where individuals cannot choose whom to interact with but they have inherently different tendencies toward forming either more positive or negative edges.

One limitation of this study is that the STP null model preserves the average signed node degree rather than the exact node degree. Consequently, it results in a “canonical ensemble” and is therefore expected to be statistically somewhat less powerful than “microcanonical” null models that preserve the signed node degree exactly (*51*). Although formulating a null model that precisely preserves signed degrees is expected to introduce a considerably more challenging combinatorial problem, it is a promising avenue that merits further investigation.

Another interesting point that would require further investigation is the appropriate statistical threshold (*z* score or *P* value). Especially in very large networks, the standard choice of ∣*z*∣ > 2 must be revisited as the tail of the distributions might deviate from a normal expectation. Going beyond triangle patterns also quickly increases the number of hypothesis tests, especially if one would consider five- or six-node patterns, potentially leading to spurious results, calling for a controlled family-wise error rate. Besides, while considering higher-order graphlets is a meaningful way to better decipher the complexity of social networks, limited by the substantially increased computational complexity, we only considered three- and four-node graphlets in this study. As an illustration of the computational complexity, within the Slashdot dataset, we encounter 571 million triangles, categorized into four distinct cases, along with 54 billion four-node graphlets that are classified into 31 distinct cases (graphlets 5 to 35 in Figs. 4 and 5).

STP randomization has widespread potential applications and extensions. To start, it can provide a more adequate alternative null model to quantify balance (*24*, *36*, *52*–*54*) or measure polarization (*2*, *4*–*6*, *8*) in social networks. Besides, the observed SB in social networks, as indicated by the STP model, shows potential to advance current sign prediction methods (*55*–*59*). In addition, the STP null model can be extended to directed and weighted networks (*60*, *61*), with the potential to contrast large-scale data against alternatives to SB, such as status theory (*1*).

Note that upon completion of our manuscript, we came across a parallel study that overlaps with our work (*62*). The presented SCM-FT null model shares the same mathematical formulation as the STP null model, apart from details of the numerical implementation, achieving consistent results at the triangle level.

## MATERIALS AND METHODS

### Signed social network datasets

The four large signed social networks analyzed in this study were downloaded from the Stanford Large Network Dataset Collection (http://snap.stanford.edu/): (i) Bitcoin-Alpha, the trust/distrust network among people who trade Bitcoin on a platform called Bitcoin Alpha; (ii) Bitcoin-OTC, the trust/distrust network among people who trade Bitcoin on a platform called Bitcoin OTC (*34*); (iii) Slashdot, friend/foe network of the technological news site Slashdot released in February 2009; and (iv) Epinions, who-trust-whom online social network of a general consumer review site Epinions. The smaller-scale Congress network is from (*32*). More details of the construction of the datasets can be found in (*1*, *33*). Network edges are considered to be undirected. This process leads to only a very limited number of edge sign inconsistencies. Such inconsistent edges are disregarded in our analysis, together with any self-loops (*52*). Only the largest connected component of each network is considered.

### Construction of the SB reference network

We create a simple model network referred to as the SB reference network, following Harary’s theorem of SB (*40*). The model network includes 120,000 nodes, on par with the largest studied datasets. Note that the primary objective of the SB reference network is not to emulate actual social networks but rather, to serve as a standardized benchmark for evaluating whether null models can successfully identify SB within a simple SB reference network.

The nodes in the SB reference network are first divided into two equal groups. We then generate two degree sequences according to power-law degree distributions with an exponent of either 2 or 3 for positive and negative degree sequences, respectively. To introduce a moderate level of correlation between these positive and negative degree sequences, we initially arranged both sequences in ascending order. Subsequently, we exchange each degree value in the positive degree sequence with another random degree value from the same sequence, with a probability of 0.2 for each exchange. The resulting positive and negative degree sequences have a correlation coefficient of 0.4. Then, the negative degree sequence is used to generate negative edges between members of different groups using the configuration model, while the positive degree sequence is used to generate positive edges between members of the same groups. The resulting SB reference network has comparable density and positive edge ratios to real-life social networks as shown in Table 1.

### Construction of the EC network

We use the edge-copying mechanism to introduce nodes into an initial network, thereby constructing a reference EC network. We used a + + + triangle as the initial condition and subsequently added nodes to the network. Each new node establishes a connection with a randomly selected node (*45*). The sign of this connection is positive with probability *q* = 0.9 to match the typical positive ratio of 0.75 to 0.92 in real networks (see Table 1). In addition, every new node connects with the neighbors of the selected node, with a probability *P* = 0.45 to match the sparsity of real networks. When a new node establishes a connection with the selected node via a positive (negative) edge, it keeps (reverses) the sign of the copied edge. The constructed EC reference network has 120,000 nodes, roughly the size of the largest studied real networks.

Apart from the EC reference network shown in the main text, we generated another two networks using different *P* and *q* values that lead to comparable density and positive ratios as real social networks (table S2). Figure S6 illustrates the degree distribution of the generated EC networks, which exhibits a rough alignment with a power-law distribution. Different EC networks yield consistent results regarding balance, as depicted in fig. S7.

### Standard null models

In the rewire null model, we randomly select two edges of four different nodes, *A* − *B* and *C* − *D*, and attempt to swap them with equal probability to either *A* − *D*, *C* − *B* or *A* − *C*, *B* − *D*. Such swap attempts are aborted if the resulting edges already exist in the network. To achieve sufficient network randomization, we perform 40 *E* edge swap attempts, where *E* is the number of edges in the network. In the signed rewire null model, we use the same method as in the rewire null model but only select edges of the same sign, thus preserving the signed degrees of each node. To prevent multi-edges with both positive and negative signs, we prohibit the swap if it would result in edges with contradictory signs.

In the sign shuffle null model, we randomly assign positive or negative signs to each edge, while preserving the exact total number of positive and negative edges.

### The STP null model

The STP null model is based on the maximum entropy framework (*27*–*31*). We extend the application of the maximum entropy framework to signed networks, enabling the simultaneous preservation of both the network topology and the signed degree sequence. A signed network *G*_0_ is first divided into two subgraphs, namely, the positive (*G*_p_) and negative (*G*_n_) subgraphs that include all the positive or negative edges in *G*_0_. Keeping the topology intact means that randomizing the negative subgraph readily provides the positive subgraph. Each negative subgraph instance *G*_nr_ is assigned a probability *P*(*G*_nr_) that maximizes the Shannon-Gibbs entropy

$$S=-\sum_{G_{\text{nr}}}\;P\left(G_{\text{nr}}\right)\;\text{ln}P\left(G_{\text{nr}}\right)$$ (2)

subject to the constraints ∑_*G*_nr__ *P*(*G*_nr_) = 1 and the average negative node degree $<k_{i}^{-}\left(G_{\text{nr}}\right)>=k_{i}^{-}\left(G_{n}\right)$ . Considering all constraints leads to the function

$$\begin{array}{c} L=S+\beta [1-\sum_{G_{\text{nr}}}\;P\left(G_{\text{nr}}\right)]+\sum_{i}\;\theta_{i}\\ {}[\langle k_{i}^{-}\left(G_{\text{nr}}\right)]-\sum_{G_{\mathrm{nr}}}\;P\left(G_{\text{nr}}\right)k_{i}^{-}\left(G_{\text{nr}}\right)\rangle \end{array}$$ (3)

where β, θ*_i_* are the Lagrange multipliers of the constraints. The solution is found by setting the derivatives of *L* with respect to *P*(*G*_nr_) and the Lagrange multipliers to 0. Solving the equations leads to *P*(*G*_nr_) = *e*^−*H*(*G*_nr_)^/*Z*, with the Hamiltonian $H\left(G_{\text{nr}}\right)=\sum_{i}\;\theta_{i}k_{i}^{-}\left(G_{\text{nr}}\right)$ and the partition function *Z* = ∑_*G*_nr__ *e*^−*H*(*G*_nr_)^. We define an element in the negative adjacency matrix as $\sigma_{\mathrm{ij}}^{-}=1$ if node *i* is negatively connected to node *j*, otherwise $\sigma_{\mathrm{ij}}^{-}=0$ . The Hamiltonian can then be expressed as

$$H=\sum_{\mathrm{ij}}\;\theta_{i}k_{i}^{-}=\sum_{i<j}\;\left(\theta_{i}+\theta_{j}\right)\sigma_{\mathrm{ij}}^{-}$$ (4)

The partition function is then

$$Z=\sum_{\left\{\sigma_{\mathrm{ij}}^{-}\right\}}\;\text{exp}[-\sum_{i<j}\;\left(\theta_{i}+\theta_{j}\right)\sigma_{ij}^{-}]=\prod_{i<j}\;[1+e^{-\left(\theta_{i}+\theta_{j}\right)}]$$ (5)

The resulting probability of selecting an existing edge in *G*_0_ to be part of *G*_nr_ between nodes *i* and *j* is simply (*63*–*65*)

$$p_{\mathrm{ij}}^{-}=\langle \sigma_{\mathrm{ij}}^{-}\rangle =\sum_{G_{\text{nr}}}\;\sigma_{\mathrm{ij}}^{-}P\left(G_{\text{nr}}\right)=\frac{1}{1+e^{\theta_{i}+\theta_{j}}}=\frac{1}{1+\alpha_{i}\alpha_{j}}$$ (6)

where we denote α*_i_* = *e*^θ_*i*_^. α*_i_* can be found efficiently, through the iterations

$${\alpha \prime }_{i}=\frac{1}{k_{i}^{-}}\sum_{j,\left(i,j\right)\in G_{0}}\;\frac{1}{\alpha_{j}+1/\alpha_{i}}$$ (7)

We use the initial condition $\alpha_{i}^{\left(0\right)}\equiv 1$ and we stop the iteration when the maximum relative change of α*_i_* is less than 10^−3^ between two consecutive iterations or it reaches the maximum number of iterations of our iterative algorithm, set to 10^4^. Note that although here we randomize the negative subgraph and set the remaining network as the positive subgraph, randomizing the positive subgraph first will give the same result.

### Empirical *P* values

We use empirical *P* values to assess the significance of the graphlet results. Specifically, we compute the one-sided empirical *P* value as *P* = (*r* + 1)/(*n* + 1), where *n* is the number of random samples, set to 1000 in this study, and *r* is the number of samples that produce a higher (lower) graphlet frequency than or equal to the observed frequency (*66*). A *P* value below 0.01 indicates that the observed graphlet frequency *n*_obs_ is significantly higher (lower) than the average graphlet frequency <*n*_rand_> in the random samples.
