# Supplementary material for: Proper network randomization is key to assessing social balance
Source: Sci Adv. 2024 May 3;10(18):eadj0104. doi: 10.1126/sciadv.adj0104 (PMC11068007; doi:10.1126/sciadv.adj0104)
Supplement: Supplementary file 1 — Supplementary Text Figs. S1 to S7 Tables S1 and S2 [file sciadv.adj0104_sm.pdf]

Science Advances
AAAS

Supplementary Materials for

Proper network randomization is key to assessing social balance

Bingjie Hao and István A. Kovács

Corresponding author: István A. Kovács, istvan.kovacs@northwestern.edu

Sci. Adv. **10**, eadj0104 (2024)
DOI: 10.1126/sciadv.adj0104

This PDF file includes:

Supplementary Text
Figs. S1 to S7
Tables S1 and S2

1 Normality of the null model samples

We checked the normality of the null model samples for the Slashdot dataset as an example. The result is shown in Fig. S1.

2 Negative benchmarks

As a negative benchmark, the SB reference network is rewired to reduce balance. We average z-scores over 10 rewired SB reference network realizations. For each realization, 100 samples were used to calculate the z-scores. All methods show no sign of SB or WB. The signed rewire and STP null models detect some graphlets with high z-scores, which could be a sign of some residual signed patterns in the randomized networks or a non-Gaussian tail in the distributions (Fig. S2A).

Another, more extreme, negative benchmark is constructed by reversing the edge signs in the SB reference network, 'SB rev'. In SB rev, nodes within the same group connect to each other with negative interactions, while they connect to nodes in the other group with positive interactions. This leads to a network with mostly unbalanced triangles and balanced squares. Note that reversing the signs in a triangle turns a balanced one into an unbalanced one and vice versa, while squares remain in the same category. Again, only the STP null model agrees with the intended structure for triangles and squares. No method claims SB or WB. For squareZs and squareXs patterns, it depends on their structure if they remain balanced (unbalanced) under reversing the signs. Again, the STP null model provides consistent results with the expectations that patterns 8, 12, 14, 21, 24-26, and 29 should be overrepresented (Fig. S2B).

3 Results for motifs

The results of squareZs and squares motifs are shown in Fig. S3.

4 Analytical results for signed triangles

We explicitly count all three- and four-node patterns for the real social networks and random samples in this study. However, the number of triangles in the STP null model can be calculated theoretically. Let A be the adjacency matrix, where $a_{ij} = 1$ if there is an edge between node i and node j, and $a_{ij} = 0$ otherwise. Let P^- be the probability matrix where p_{ij}^- is the probability of a negative edge between node i and node j calculated in equation (6) of the main text. P^+ is then the probability matrix where node i a ode j are connected by a positive edge with

probability $p_{ij}^+ = a_{ij} - p_{ij}^-$. Then, the number of $---$ triangles is calculated as

$$\begin{aligned} T^{---} &= \frac{1}{6}\sum_{i=1}^{N}[P^- P^- P^-]_{ii} \\ &= \frac{1}{6}\sum_{i=1}^{N}\sum_{\substack{j=1\\(j\neq i)}}^{N}\sum_{\substack{k=1\\(k\neq i,j)}}^{N} p_{ij}^- p_{jk}^- p_{ki}^- \\ &= \frac{1}{6}\sum_{i=1}^{N}\sum_{\substack{j=1\\(j\neq i)}}^{N}\sum_{\substack{k=1\\(k\neq i,j)}}^{N} \frac{1}{(1+\alpha_i\alpha_j)(1+\alpha_j\alpha_k)(1+\alpha_k\alpha_i)}, \end{aligned} \tag{S1}$$

where 6 accounts for the number of permutations of the three nodes in a triangle. However, for this exact formula to be useful, in general, α_i must be first determined numerically. In the limit of $\alpha_i >> 1$ ('sparse' approximation when the negative node degree k^- is small), the connection probability can be approximated by the configuration model with $\alpha_i \sim \frac{\sqrt{2m^-}}{k_i^-}$, where m^- is the number of negative edges. With this approximation, in the $N \gg 1$ limit, the number of $---$ triangles reduces to

$$T^{---} \approx \frac{1}{6}\sum_{i=1}^{N}\sum_{j=1(j\neq i)}^{N}\sum_{k=1(k\neq i,j)}^{N} \frac{(k_i^-)^2(k_j^-)^2(k_k^-)^2}{(2m^-)^3} \approx \frac{1}{6}\left(\frac{K^-}{2m^-}\right)^3, \tag{S2}$$

where $K^- = \sum_i (k_i^-)^2$. The number of $+++$, $++-$, and $+--$ triangles can be calculated similarly as

$$T^{+++} = \frac{1}{6}\sum_{i=1}^{N}[P^+ P^+ P^+]_{ii} = \frac{1}{6}\sum_{i=1}^{N}\sum_{\substack{j=1\\(j\neq i)}}^{N}\sum_{\substack{k=1\\(k\neq i,j)}}^{N} p_{ij}^+ p_{jk}^+ p_{ki}^+ \approx \frac{1}{6}\left(\frac{K^+}{2m^+}\right)^3, \tag{S3}$$

$$T^{++-} = \frac{1}{2}\sum_{i=1}^{N}[P^+ P^+ P^-]_{ii} = \frac{1}{2}\sum_{i=1}^{N}\sum_{\substack{j=1\\(j\neq i)}}^{N}\sum_{\substack{k=1\\(k\neq i,j)}}^{N} p_{ij}^+ p_{jk}^+ p_{ki}^- \approx \frac{1}{2}\left(\frac{K^*}{2m^+}\right)^2 \frac{K^+}{2m^-}, \tag{S4}$$

$$T^{+--} = \frac{1}{2}\sum_{i=1}^{N}[P^+ P^- P^-]_{ii} = \frac{1}{2}\sum_{i=1}^{N}\sum_{\substack{j=1\\(j\neq i)}}^{N}\sum_{\substack{k=1\\(k\neq i,j)}}^{N} p_{ij}^+ p_{jk}^- p_{ki}^- \approx \frac{1}{2}\left(\frac{K^*}{2m^-}\right)^2 \frac{K^-}{2m^+},, \tag{S5}$$

where $K^* = \sum_i k_i^+ k_i^-$, $K^+ = \sum_i (k_i^+)^2$, and m^+ is the number of positive edges. As an example, the approximate analytical results of triangles are shown in Table S1 for the Congress dataset. While there are some deviations due to the approximations, the results are qualita-

tively consistent with the explicit counts of triangles. Larger patterns may also be calculated analytically, for example, the number of $++++$ square motifs can be calculated as

$$S^{++++} = \frac{1}{24}\sum_{i=1}^{N}[P^+P^+P^+P^+]_{ii} = \frac{1}{24}\sum_{i=1}^{N}\sum_{\substack{j=1 \\ (j\neq i)}}^{N}\sum_{\substack{k=1 \\ (k\neq i,j)}}^{N}\sum_{\substack{l=1 \\ (l\neq i,j,k)}}^{N} p^+_{ij}p^+_{jk}p^+_{kl}p^+_{li} \approx \frac{1}{24}\left(\frac{K^+}{2m^+}\right)^4. \tag{S6}$$

5 Edge copying mechanism

5.1 Illustration of edge copying mechanism

In the main text, we illustrate the edge copying mechanism with a simple example of three nodes. Here we show that with four nodes, the edge copying mechanism leads to all possible balanced squareZs and squareXs (Fig. S4).

5.2 Theoretical proof of balance in the EC reference network

We illustrate the mechanism of creating an EC reference network in Fig. S5 with a network initially containing a balanced triangle. Two nodes (node 4 and 5) are added to the network sequentially and copy the same node (node 2). Within the first two steps of edge copying, we observe all three- and four-node graphlets of interest. We define the sign of a triangle as the products of its edge signs

$$S_{triangle} = s_i s_c s_{c'} = s_i s_c (s_i s_c) = s_i^2 s_c^2 > 0 \tag{S7}$$

where s_i denotes the sign of the edge connecting the added node and the node that is being copied and s_c ($s_{c'}$) denotes the sign of the edge that is being copied (copied). As triangles are always balanced, squareZs and squareXs formed by combinations of triangles must also be balanced. However, the balance of squares is not explicitly guaranteed by balanced triangles. The highlighted diagram in Fig. S5 shows the mechanism that generates squares. The sign of the square $1-3-4-5$ is determined as

$$S_{square} = s_{13}s_{34}s_{45}s_{15} = s_{13}(s_{23}s_{24})(s_{25}s_{24})(s_{12}s_{25}) = (s_{12}s_{13}s_{23})s_{24}^2 s_{25}^2. \tag{S8}$$

With triangles always balanced as defined in equation (S7), squares are also always balanced. Unlike the node copying mechanism, the edge copying mechanism could form all possible balanced squares, including $+-+-$. Considering two nodes copying different nodes or further steps of node addition follow analogous conclusions, and eventually lead to a network with only balanced patterns.

5.3 Properties of the EC reference network

The overview of the EC reference network is shown in Table S2. The EC reference network is generated with three different parameter sets, EC12_1, EC12_2, and EC12_3. The positive ratio is defined as the ratio of positive edges to the total number of edges. The signed node degree distribution is shown in Fig. S6.

5.4 Results of the EC reference network

The results of the EC reference networks of different configurations are shown in Fig. S7.

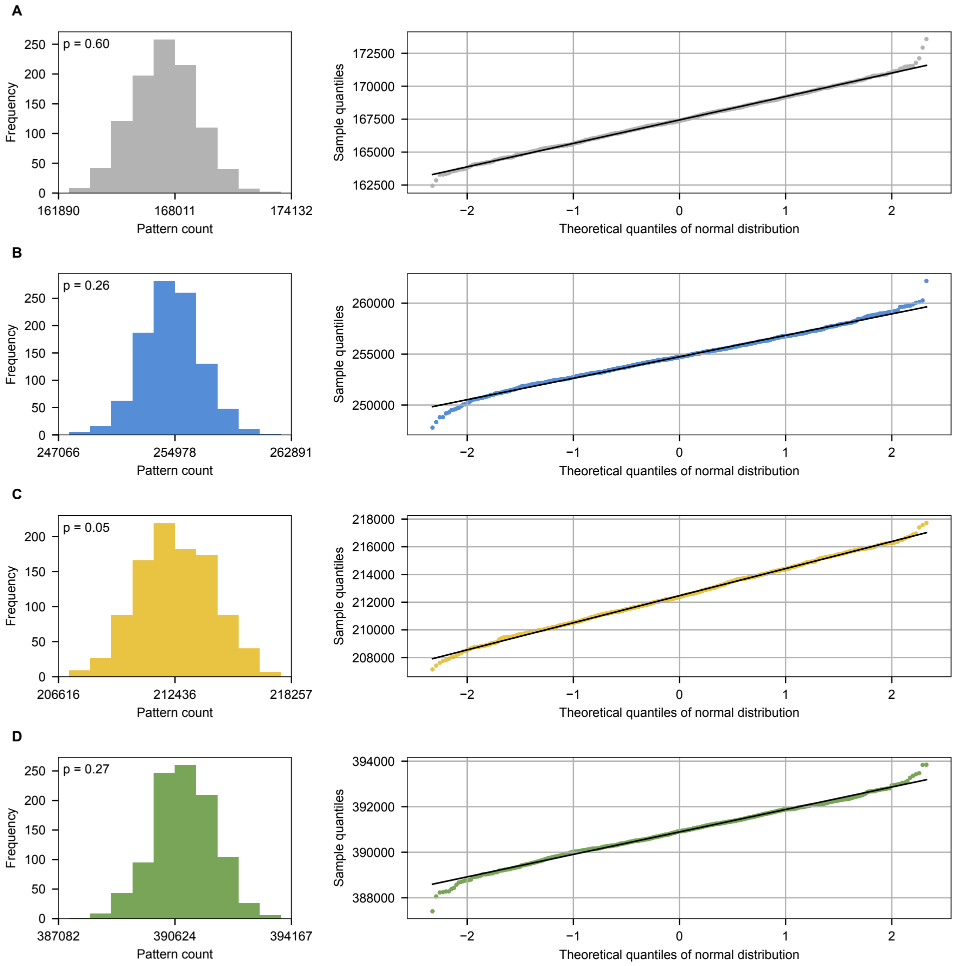

Figure S1: Normality of the null model samples for the Slashdot dataset shown for the $+++$ triangle as an example. Left: The distribution of frequencies of the $+++$ triangle in $n = 1000$ (A) rewire (B) sign shuffle (C) signed rewire (D) STP random samples. In most cases, we see $p > 0.05$, meaning that we fail to reject the null hypothesis that the data are normally distributed. Note that there are some instances where the test suggests potential non-normality. In these cases, the z-scores should be interpreted cautiously along with the empirical p-values, as the normality assumption may be violated. Right: The Q-Q plot of frequencies of the $+++$ triangle in $n = 1000$ (A) rewire (B) sign shuffle (C) signed rewire (D) STP random samples. The data points on the Q-Q plot roughly follow a straight line, suggesting that the bulk of the data is normally distributed.

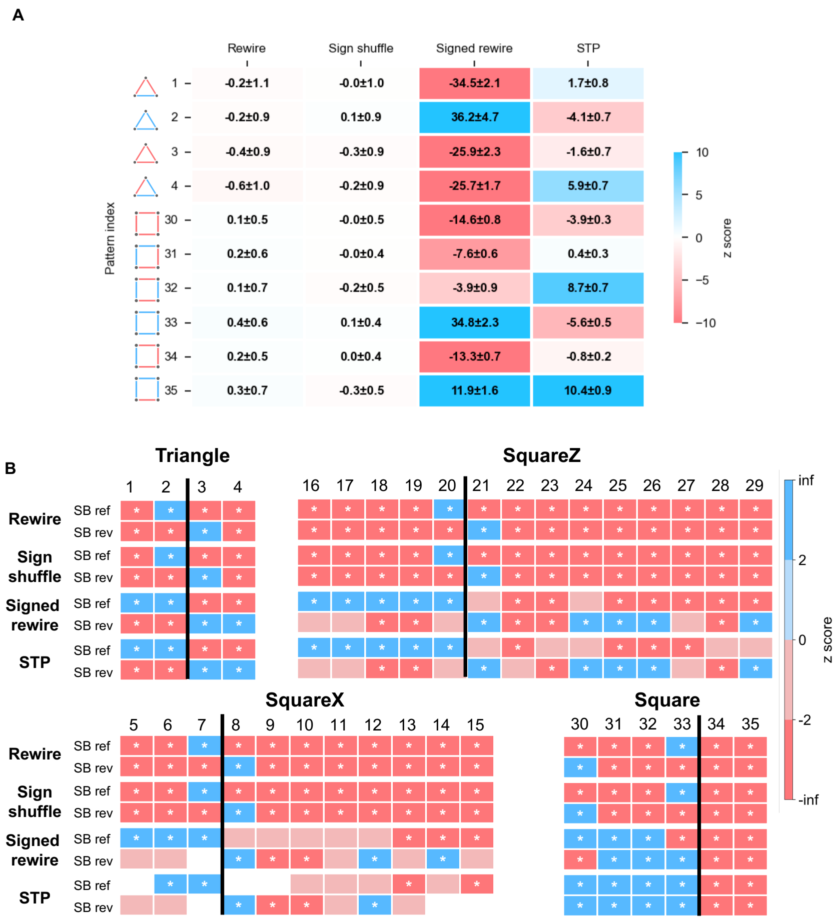

Figure S2: Negative benchmarks. (A) Results for rewired SB networks. z-scores are indicated as mean $\pm$ SD. (B) No SB or WB was observed at the triangle, squareZ and squareX levels in the SB rev network (where edge signs are reversed).

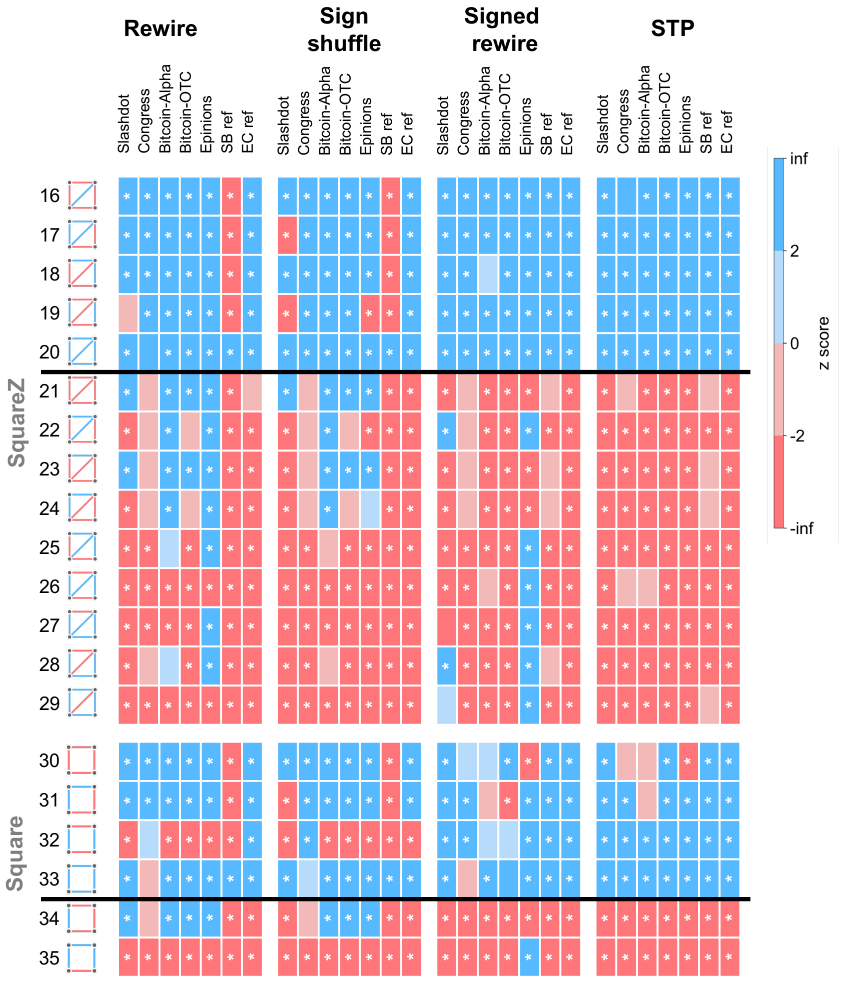

Figure S3: Comparison of squareZ and square motif significance in the studied networks. The z-scores are indicated by blue (overrepresented) and red (underrepresented) blocks. We first list the balanced motifs according to SB, separated by a black line from the unbalanced motifs. We leave the block white if $\sigma_{rand} = 0$ since it leads to an undetermined z-score. Significant results with both $|z| > 2$ and $p < 0.01$ are indicated by *. The statistical analysis is performed using a sample size of $n = 1000$.

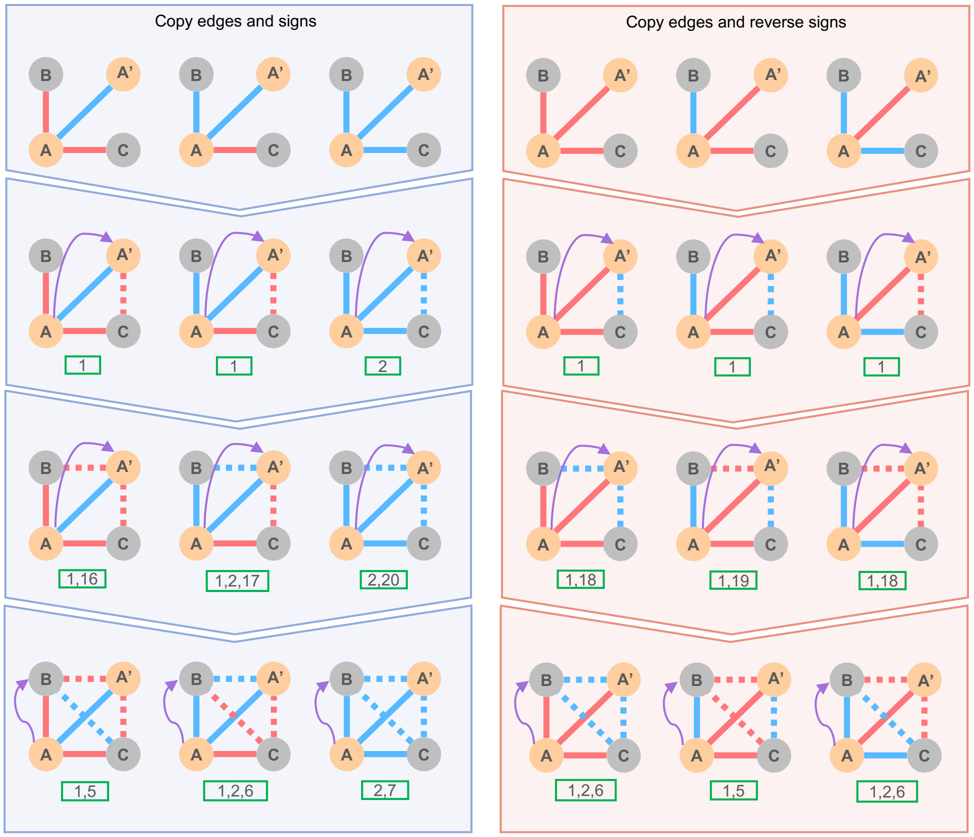

Figure S4: Illustration of the edge copying mechanism with four nodes. The edge copying mechanism is illustrated with a network initially containing four nodes. With nodes A' copying node A, balanced triangles and squareZs are formed. It eventually leads to squareXs when other nodes start to copy each other, for example node B copying node A. Positive and negative edges are indicated by blue and red lines, respectively. The dashed lines indicate the copied edges.

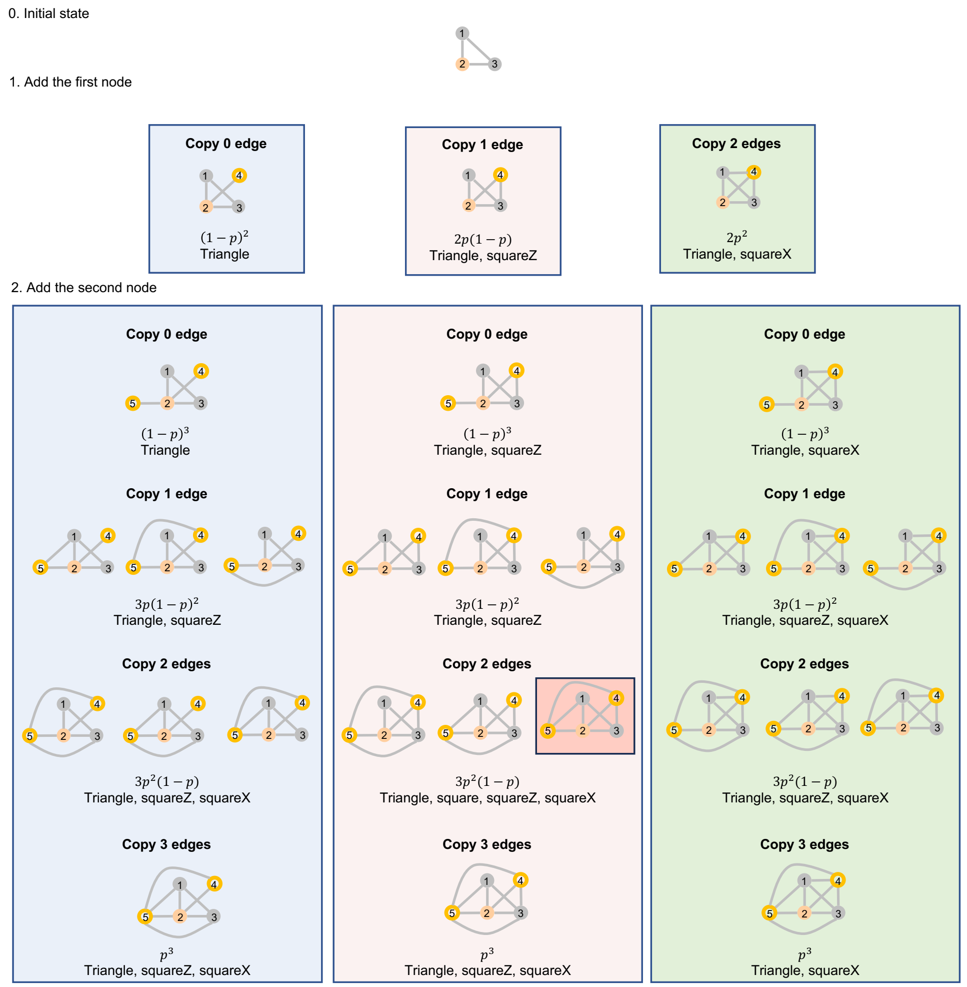

Figure S5: The first two steps of the EC reference network construction. The edge copying mechanism is illustrated with a network initially containing a balanced triangle. Two nodes (node 4 and 5) are added to the network sequentially and copy the same node (node 2). The highlighted diagram shows the mechanism that generates square graphlets. For simplicity, signs are not shown in the figure.

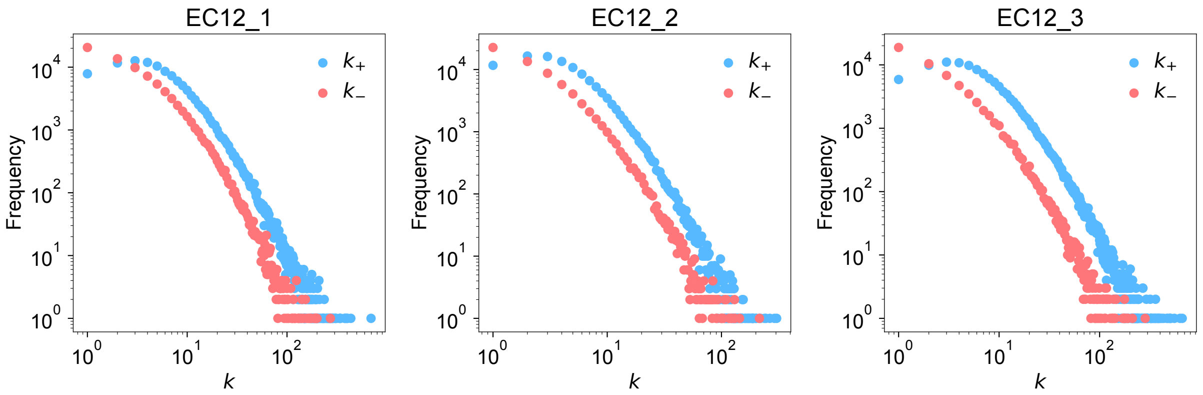

Figure S6: Positive and negative degree distributions of three variants of the EC reference network.

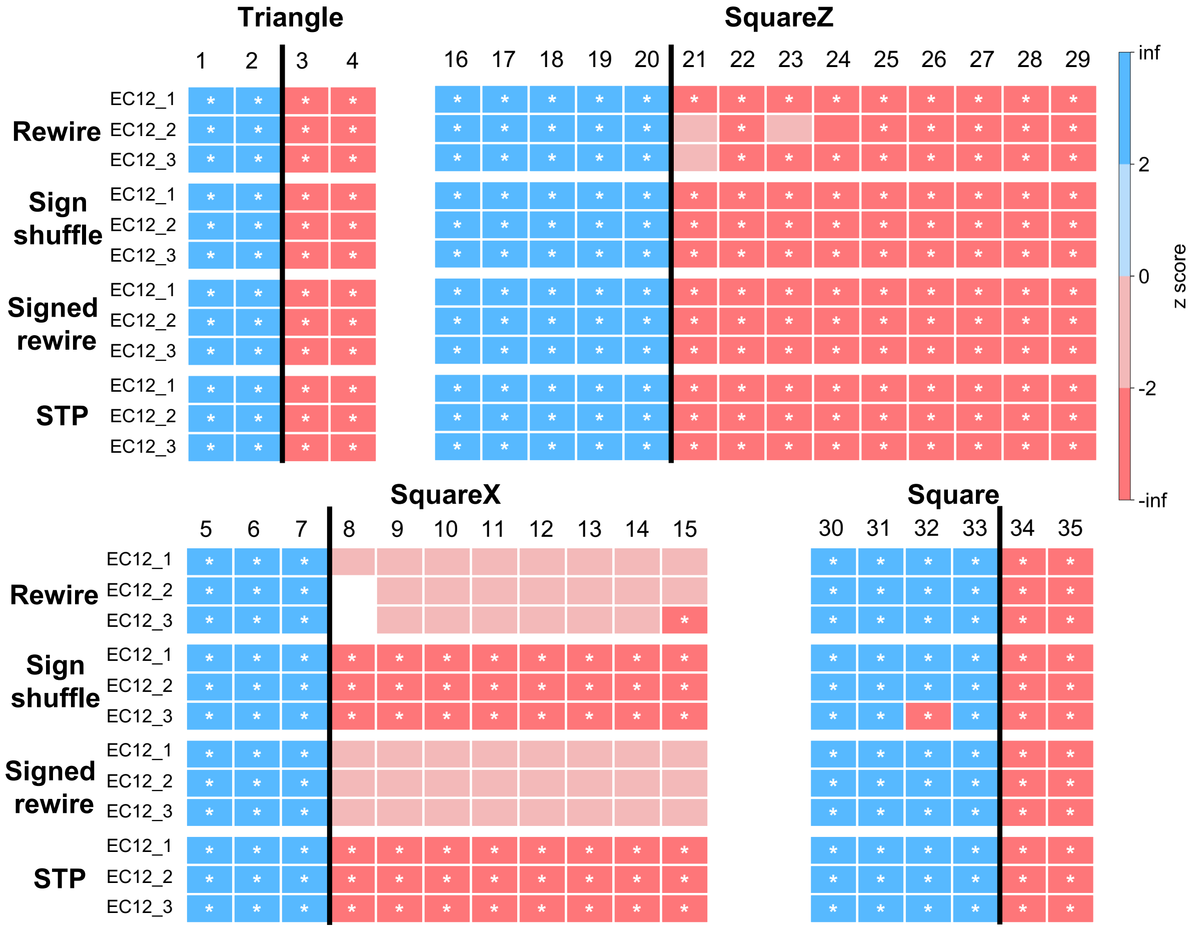

Figure S7: Overview of graphlet significance in EC reference networks. The z-scores are indicated by blue (overrepresented) and red (underrepresented) blocks. Balanced graphlets are listed first, separated from the unbalanced ones by a black line. Significant results with $p < 0.01$ and $|z| < 2$ are marked by *. The statistical analysis is performed using a sample size of $n = 1000$.

Table S1: Comparison between triangle numbers obtained through explicit counting and the analytic approximation for the Congress dataset.

Triangle	Explicit count	Approximate formula
$---$	8	10
$+++$	99	126
$++-$	66	70
$+--$	35	30

Table S2: Overview of the generated EC reference networks.

Dataset	EC12_1	EC12_2	EC12_3
p	0.45	0.40	0.45
q	0.90	0.90	0.95
Nodes	120,000	120,000	120,000
Edges	790,591	547,314	853,466
Density	0.00011	0.00008	0.00012
Positive ratio	0.72319	0.74393	0.82321
